# Short-Term Impacts of a School-Based Teen Pregnancy Prevention Program for Latino Youth: a Cluster Randomized Trial

**DOI:** 10.1007/s11121-025-01805-y

**Published:** 2025-04-14

**Authors:** Krystle McConnell, Sahra Ibrahimi, Martha Yumiseva, Salwa Shan, Amy Lewin

**Affiliations:** 1 Department of Health Sciences, Towson University, 8000 York Road, Towson, MD 21252 USA; 2 Department of Global Health, Denison University, Granville, OH 43023 USA; 3 Department of Family Science, University of Maryland, College Park, MD 20742 USA

**Keywords:** Teen pregnancy prevention, Latino youth, Randomized controlled trial

## Abstract

This study evaluates El Camino, a goal-setting sexual health promotion program developed for Latino youth. A cluster randomized controlled trial was conducted in grades 9–12 in 68 classrooms (*n* = 746 students) across 11 schools in Maryland with large populations of Latino students. A total of 208 students were lost to follow-up, leaving 538 students for an intention-to-treat analysis (El Camino, 34 clusters, *n* = 289; control, 34 clusters, *n* = 249). At baseline, most participants (72.1%) reported never having had vaginal sex and no sex in the last 3 months (84.4%). This study did not detect any significant intervention impacts on sexual behavior outcomes but found impacts on several proximal sexual health outcomes. At post-test, approximately 1–2 weeks after curriculum completion, scores were significantly higher among El Camino participants compared to control participants on measures of contraception knowledge (*β* = 0.5 (cluster robust SE 0.1)), condom knowledge (0.4 (0.1)), consent knowledge (0.3 (0.1)), and awareness of birth control methods (0.9 (0.1)), as well as confidence to discuss sex with a partner (0.3 (0.1)). A total of 84.7% of El Camino participants reported positive attitudes toward condom use compared to 67.1% of control participants (OR = 2.7, 95% CI [1.8, 4.0]); 83.1% reported intending to use condoms if sexually active compared to 72.6% of control participants (1.9, [1.2, 2.9]); 80.9% reported knowing where to get birth control compared to 48.9% of control participants (4.4, [2.8, 7.0]), and 83.3% reported being confident to state and ask for consent compared to 70.5% of control participants (2.1, [1.2, 3.7]). This study also found that intervention impacts varied by student gender and program implementation factors. Overall, El Camino appears to be effective in improving sexual health knowledge, self-efficacy, and intentions among Latino youth.

Trial registration: Clinicaltrials.gov NCT06485284. Date 7–3 - 24.

**Supplementary Information:**

The online version contains supplementary material available at 10.1007/s11121-025-01805-y.

Unintended teen pregnancy has been identified as a public health issue because it is associated with increased risks for a range of health and social adversities, including preeclampsia, low birthweight, prematurity (Akinbami et al., 2000), and symptoms of depression, child behavior problems, maladaptive parenting, and child maltreatment (Cygan et al., 2020; Lewin et al., 2013; Phipps & Nunes, 2012). Additionally, teen childbearing may limit or delay educational attainment and potentially harm future employment opportunities, leading to decreased income and a higher likelihood of living in poverty (Fuller et al., 2018; McClay & Moore, 2022; Rosenthal et al., 2009). Importantly, not all teen pregnancies are unintended, and not all teen parents have poor outcomes (Hans & White, 2019). However, being a teen parent elevates the risk for these outcomes relative to peers who delay childbearing, particularly among those whose early childbearing is unintended.

According to the 2023 Youth Risk Behavior Survey (YRBS), 31.6% of high school students in the USA. reported having ever engaged in sexual intercourse (CDC, 2023). Although Latino youth are not having sexual intercourse at higher rates than other groups, they are more likely than White and Asian youth to report not using any form of contraception the last time they had intercourse with an opposite-sex partner (15.4% of Latino youth vs. 8.3% of White youth and 10.4% of Asian youth). Overall rates of teen childbearing have steadily decreased since the 1990s, a trend largely attributed to greater access to comprehensive sexual health education, improved availability of reproductive health care, and declining rates of heterosexual vaginal intercourse among adolescents (Lindberg et al., 2021; Osterman et al., 2021). Despite this overall decline, rates of teen childbearing among Latino youth have decreased less significantly compared to other demographic groups and remain disproportionately high (Morales-Alemán & Scarinci, 2016). Latino youth have a higher likelihood of early childbirth, with 9% giving birth by age 18, compared to 4% of White teenagers and 8% of Black teenagers (Abma & Martinez, 2023).

There are many systemic factors that may contribute to the relatively high percentage of teen births in the Latino population in the United States. This group of youth experiences numerous social disparities that may increase their risk for unintended teen pregnancy: they are more likely to be living in poverty, they may lack access to health insurance and reproductive health services, and they may have lower educational support when it comes to reproductive health and contraception (Rocca et al., 2010; Xiao et al., 2023). Several sociocultural factors, including religious and cultural stigma surrounding reproductive health care and contraception use, and social norms around sexual initiation and early childbearing among Latino communities, may also contribute to an increased risk for unintended teen pregnancy (Aparicio et al., 2016).

School-based teen pregnancy prevention programs are especially promising to reduce disparities in unintended teen pregnancy, as they provide more universal access for youth. However, findings have largely been mixed, possibly due to varying participant characteristics, such as race/ethnicity and gender composition, as well as implementation moderators, such as program setting and program delivery personnel (Juras et al., 2019, 2022; Vasilenko et al., 2023). Recent evaluations of school-based teen pregnancy programs have been more promising. For example, a cluster-randomized trial evaluating Making Proud Choices! found positive effects on risk and protective factors (e.g., knowledge, self-efficacy, attitudes) known to be associated with sexual behavior change, as well as decreased sexual activity in the last 3 months (Cole et al., 2024). A cluster randomized trial evaluating the impact of *Re:Mix*, a school-based pregnancy prevention program, found positive intervention effects on risk and protective factors among a sample of predominantly Latino youth (Manlove et al., 2021). Yet few school-based sexual health programs have been designed to be culturally responsive to the needs of Latino youth specifically. While programs such as Familias Unidas (Familias Unidas, n.d.) and ¡Cuídate! (Farb et al., 2018) provide extensive services, including substance abuse and mental health support in addition to sexual health promotion, their outreach is limited and does not cover all Latino teenagers across the country (Familias Unidas, n.d.). To reduce disparities in unintended teen pregnancy and sexual health more generally, there is a need for more school-based programming that is culturally responsive and engaging. El Camino is a goal-setting sexual health promotion program, based on principles of positive youth development, that was created specifically for Latino youth (Child Trends, 2022).

In preparation for the development of this program, researchers conducted focus groups and interviews separately with Latino parents and youth to gather information about the needs and concerns of both parents and teens. Findings from these initial interviews allowed researchers to incorporate feedback into the curriculum such as addressing barriers to communication, allowing for opportunities to discuss real-life scenarios and consequences, and highlighting important sources of support in teen’s lives so they can gain knowledge about sexual and reproductive health. After these components were incorporated, activities were pilot tested with Latino youth, and researchers debriefed to further gain insights into potential improvements and to learn about how activities were received by Latino youth. Ultimately, this led to the creation of a curriculum consisting of eleven 45-min lessons, divided into three sections, that encourage youth to set goals, make informed decisions about their sexual and reproductive health, and promote healthy relationships.

The program was implemented in 11 Maryland schools and taught in either English or Spanish (Child Trends, 2021). Three of the implementation sites took place at high schools with school-based health and wellness centers (wellness center schools). Identity, Inc., a community-based positive youth development organization, operates these wellness centers and has established relationships with participating schools. Staff from Identity facilitated the intervention across all schools. Implementation of the program for the first cohort was virtual due to the pandemic, but programming for the remaining cohorts was delivered in person.

The natural structure of students nested within classrooms provides the basis of the cluster randomized trial design. The purpose of the current study is to evaluate the short-term impacts of El Camino at the individual student level, answering the following research questions: (1) Whether participation in El Camino is associated with less sexual behavior risk (measured as avoiding penile-vaginal sex without any method of contraception, avoiding penile-vaginal sex without a condom, or avoiding penile-vaginal sexual activity) for the intervention group relative to a control group; (2) whether participation in El Camino is associated with key precursors of healthier sexual behavior, including increased knowledge related to sexual and reproductive health, increased self-efficacy to practice health-promoting behaviors, more positive attitudes around sexual and reproductive health, and greater intentions to practice sexual health at post-intervention relative to a control group; and (3) whether the impacts of the intervention vary by contextual factors, including gender, if the groups were conducted virtually or school type (wellness center vs. non-wellness center). This evaluation extends previous research on school-based teen pregnancy prevention efforts by assessing the impact of a program for a unique sample of largely recent immigrant and Spanish-speaking youth and examining whether the impacts differ based on implementation characteristics.

## Methods

### Participants

The study is an unblinded cluster randomized controlled trial enrolling youth in the 9 th- 12 th grades in 68 classrooms across eleven public schools with large Latino student populations in a large urban/suburban county in Maryland. El Camino was implemented across six cohorts of students between spring 2021 and fall 2022, including two summer cohorts. Youth attending one of the targeted partner schools who had not taken the El Camino curriculum previously were eligible for the evaluation. Student assent and parental consent were obtained prior to enrollment in the evaluation.

Identity staff at each school recruited students to participate in one of two groups within a classroom. After students completed the baseline survey, their classrooms (cluster) were randomly assigned to receive the El Camino program or an unrelated program of the same length (45-min lessons delivered weekly across ~ 11 weeks) by the independent evaluator. Mathematica, which was contracted by the Office of Population Affairs to provide Evaluation Technical Assistance on the Teen Pregnancy Prevention grants, provided consultation on randomization allocation each semester to ensure that classrooms were balanced both within and across semesters. Classrooms were eligible for randomization as long as five eligible students were enrolled. Each curriculum was delivered in either English (25.0%) or Spanish (75.0%), depending on the language preferences of the group. Simple randomization was stratified across semesters to achieve a balance of English and Spanish groups.

Prior to carrying out the study, we conducted power analyses to determine the minimum detectable impact (MDI) for two measures of sexual abstinence and two measures of unprotected sex. These MDIs were based on classroom-level random assignment, with an 80% level of statistical power, 5% alpha, 80% consent rate, and an anticipated follow-up response rate of 80%. MDIs were based on an intraclass correlation coefficients (ICC) of 0.04, based on previous research examining school- and classroom-based ICC levels for reproductive health outcomes. We used national data on Hispanic 11 th graders from the 2017 Youth Risk Behavior Surveillance Surveys (YRBS) to estimate levels of sexual experience, sexual activity, and unprotected sex at the time of a 1-year follow-up for our proposed sample. Based on these assumptions, our power analyses indicated that, with 60 classes of 20 consented students and a sample of 960 students after 20% attrition, the proposed sample size would be sufficient to detect impacts between five and 10 percentage points, depending on the outcome.

**Fig. 1:**
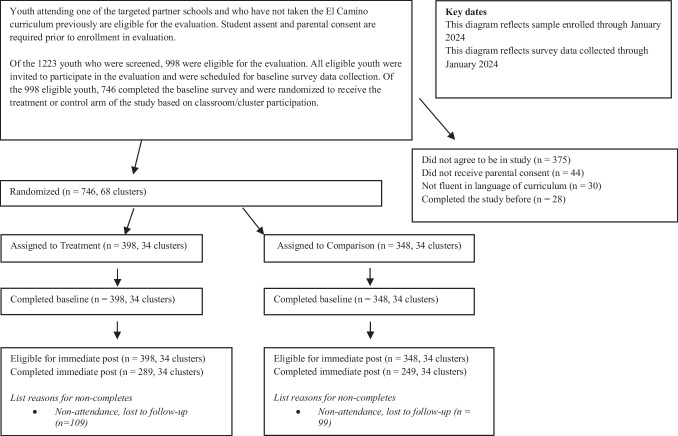
Consort diagram for youth, El Camino program

The evaluation recruited 998 eligible students across participating schools. Of the identified students, 252 (25.3%) did not agree to be in the study, did not receive parental consent, or had previously completed the pilot study. Ultimately, 746 students (74.7%) were enrolled and were nested in 68 classrooms, 34 of which were randomly assigned to receive the El Camino curriculum and 34 of which were randomly assigned to receive the control curriculum, after accounting for the language preference of the participating students. All enrolled students took the baseline survey (*n* = 746), and 538 (72.1%) completed the immediate post-test survey (34 El Camino clusters, *n* = 289; 34 control clusters, *n* = 249). See the consort diagram for full enrollment and attrition details at the cluster and youth levels (Fig. 1).

### Instrumentation

Student baseline and post-test surveys obtained information on (1) sexual behaviors, including whether participants ever had penile-vaginal sex, whether participants had penile-vaginal sex in the last 3 months, whether participants had unprotected penile-vaginal sex without any method of contraception in the last 3 months, and whether participants had unprotected penile-vaginal sex with no condom in the last 3 months (primary outcomes); (2) intentions, including whether participants intended to use contraception and whether participants intended to use condoms if sexually active in the next year; (3) knowledge, including awareness of birth control methods, condom knowledge, contraception knowledge, and consent knowledge; (4) attitudes toward condoms and birth control, and (5) self-efficacy, including knowing where to get birth control, confidence in going to a clinic to get contraception, confidence in discussing sex and contraception with a partner, confidence in setting limits with a partner, and confidence in stating and asking for consent (secondary outcomes). See supplemental Appendix A for a full list of outcome measures, source questions, and operationalization of outcome measures. See this brief for further details on survey source questions, survey item development, and outcome measurement development (Welti & Faccio, 2020).

### Procedure

Intervention: El Camino is a school-based goal-setting teen pregnancy prevention program that encourages youth to set goals, make informed reproductive health choices, and have healthy relationships (See Child Trends, 2021; Child Trends, 2022 for more details). The El Camino curriculum is divided into three sections or “arcs”: (1) goal setting, (2) sexual and reproductive health, and (3) communication and healthy relationships. The curriculum has a cross-cutting focus on goal setting, and it includes lessons and activities to help students delay sexual activity and avoid risky sexual behaviors. The program employs interactive group activities, including role plays and reflective discussion, that encourage students to identify their viewpoints and discuss why they feel the way they do. Participating teens also read and discuss a series of novelas (short stories) about teens in relatable situations, which help them think through issues around consent, healthy relationships, and reproductive health that they may encounter in their daily lives. The curriculum was originally intended to be delivered in person; however, the curriculum was adapted for virtual delivery in Spring 2021 due to the COVID- 19 pandemic. Implementation during the Spring semester 2021 was virtual and then was in person for subsequent cohorts, delivered either during a lunch period or after school (Faccio et al., 2023; Parekh et al., 2021). Control curricula: Control groups received one of several control curricula. The largest number of control groups received PODER (*n* = 28), an 11-session curriculum that focuses on youth leadership development. Other control groups received Encuentros (*n* = 3), a psychoeducational curriculum focused on mental health, El Joven Noble (*n* = 2), a youth development and leadership curriculum, and Courageous Queens (*n* = 1), a socio-emotional and character strengthening curriculum. PODER was the only control curriculum delivered virtually in Spring 2021. As with the El Camino groups, all subsequent groups were delivered in person in schools. None of the control curricula included content related to sexual and reproductive health, healthy relationships, or goal setting.

### Data Collection

The evaluation team, in partnership with facilitators, administered the baseline survey to enrolled students approximately 1–2 weeks prior to curriculum implementation. The post-test survey was administered to students approximately 1–2 weeks after curriculum completion. Survey data were collected either virtually or in person depending on context and respondent preference. All surveys were completed and stored in REDCap using unique survey links and a unique ID for each participant (Harris et al., 2009, 2019). Surveys were completed primarily on school-provided laptops or computers so responses could be kept private. Individual links to the electronic survey were distributed to students either through private chat when on Zoom or by school email when in person. All baseline surveys were administered in a group setting, where a facilitator read the questions and response options aloud while participants completed their questionnaires individually on their devices. Follow-up surveys were distributed either in groups or individually to participants who did not attend one of the group sessions. All data were maintained to preserve confidentiality and were de-identified prior to analysis. All study procedures were approved and monitored by the Child Trends Institutional Review Board.

### Data Analysis

We used an intent-to-treat approach in the analysis. All analyses were done in SAS Studio (V. 3.81). We employed generalized estimating equation models, using a repeated statement to appropriately account for clustering, to assess the following: (1) whether student characteristics differed between the intervention and control groups at baseline, (2) whether there was any differential attrition based on intervention group, and (3) whether El Camino had a positive impact on outcomes of interest at post-test. Binomial regression models (link = logit) were used to estimate impacts on dichotomous outcomes, including sexual behavior outcomes, while linear regression models (link = identity) were used to estimate impacts on non-dichotomous proximal outcomes (Manlove et al., 2021; Rotz et al., 2016). Complete case analysis with regression adjustment for baseline covariates was used in the analysis. Because the sample size varied across outcomes, we ran sensitivity tests to assess baseline equivalence for each analytic sample used in the analysis. For each outcome assessed, models included an indicator for the intervention group and were adjusted for the baseline value for the outcome, sociodemographic characteristics (age, length of time in the US, Hispanic ethnicity, gender identity, sexual orientation, language spoken at home), and school fixed effects. Gender refers to the current gender that the student identifies as, independent of anatomical sex.

To better understand differences in intervention impacts depending on context, we ran a series of moderation analyses to assess whether gender (self-identified female, male), school type (schools that do or do not include a school-based wellness center), and implementation type (virtual vs. in-person) moderated the associations between intervention and each outcome assessed. Intervention × moderator interaction terms were included in moderation models along with main effects. When the interaction term was significant (*p* < 0.05), stratified intervention effects were estimated in separate models. Standardized beta coefficients and adjusted means for continuous outcomes, as well as odds ratios and adjusted prevalence for dichotomous outcomes were estimated for all intervention effect measures.

## Results

### Participant Characteristics

Demographic characteristics are presented in Table 1. Participants in the program were, on average, 16 years old (SD = 1.5), predominantly Hispanic/Latino (83.5%), and born outside of the US (78.8%), with a mean length of time in the US of 2.5 (SD = 2.5) years. Compared to the control group, participants in the El Camino intervention group were more likely to be female (59.4% vs. 49.2%) and less likely to report only heterosexual attraction (65.3% vs. 75.0%). Full outcome characteristics at baseline are presented in supplemental Appendix B. Overall, sexual activity in this sample was low. Most participants (72.1%) reported never having had penile-vaginal sex at baseline, and only 15.6% of the sample reported having had such sex in the last 3 months. There were no differences in any of the outcome measures at baseline between the intervention and control groups. Attrition among the full sample was low and comparable within both the intervention arm (27.4%) and the control arm (28.4%) (*p* = 0.7). Therefore, the threat of non-response bias is low.

**Table 1:** Baseline demographic characteristics by treatment condition for sexual behavior analytic sample (*n* = 448)

Baseline measure	Mean (SD) or %			Difference (mean or %)	Difference (SD units)	*p*-value
	El Camino *n* = *243*	Control *n* = *205*	Total *N* = *448*			
Age	16.3 (1.5)	16.4 (1.5)	16.3 (1.5)	− 0.1	− 0.14	0.32
Hispanic	84.2%	82.8%	83.5%	1.4%	− 0.03	0.51
Gender identity						
Female	59.4%	49.2%	54.7%	10.2%	0.21	0.04
Male	39.5%	48.8%	43.8%	− 9.3%	− 0.18	0.08
Other	1.1%	2.0%	1.5%	− 0.9%	− 0.08	0.37
Length of time in US						
Born in the US	24.2%	18.9%	21.2%	5.3%	0.13	0.64
< 3 years	43.7%	53.1%	48.0%	− 9.4%	− 0.09	0.31
≥ 3 years	29.5%	25.9%	27.8%	3.6%	0.08	0.53
Language						
Mostly Spanish	63.4%	75.0%	68.8%	− 11.6%	− 0.25	0.09
Mostly English	12.5%	8.5%	10.7%	4.0%	0.13	0.27
Both Spanish and English	22.3%	14.9%	18.9%	7.4%	0.19	0.07
Other	1.7%	1.6%	1.7%	0.1%	0.01	0.89
Sexual attraction						
Only heterosexual	65.3%	75.0%	69.8%	− 9.7%	− 0.21	0.02
Only homosexual	1.4%	1.2%	1.3%	0.2%	0.02	0.85
Other	32.5%	23.3%	28.9%	9.2%	0.21	0.02
Never had sex	71.7%	72.5%	72.1%	− 0.8%	− 0.02	0.72

### Outcomes

Adjusted estimates (OR [95% CI] or standardized *β* coefficients [SE]) and adjusted prevalence or means by intervention arm are presented in Table 2. Intraclass correlation coefficients for each outcome assessed are also presented in Table 2 and ranged from − 0.06 to 0.01.

**Table 2:** Adjusted impact analysis of sexual health outcomes by treatment condition (*n* = 396–448)

Outcome	Analytic sample size		ICC	Estimate OR (95% CI) or (SE)	Adjusted prevalence (%) or adjusted means (SE)		Difference (% or scale pts)	*p*-value
	El Camino	Control	*N* = 68 clusters		El Camino	Control		
Sexual behavior								
Never had sex	*n* = *243*	*n* = *205*	− 0.04	0.7 (0.4, 1.3)	75.1%	81.0%	− 5.9%	0.25
No sexual activity in the last 3 months	*n* = *240*	*n* = *204*	− 0.03	1.0 (0.5, 1.7)	76.2%	76.8%	− 0.6%	0.90
Sex in the last 3 months without a method of contraception	*n* = *230*	*n* = *196*	− 0.001	1.4 (0.6, 2.9)	9.0%	6.7%	2.3%	0.42
Sex in last 3 months without a condom	*n* = *231*	*n* = *194*	0.02	2.0 (0.7, 5.6)	10.5%	5.5%	5.0%	0.17
Intentions								
Intend to use condoms	*n* = *254*	*n* = *218*	− 0.03	1.9 (1.2, 2.9)	83.1%	72.6%	10.5%	0.01
Intend to use contraception	*n* = *254*	*n* = *218*	− 0.02	1.4 (1.0, 2.0)	65.7%	58.1%	7.6%	0.08
Knowledge								
Knowledge about birth control (# correct, 0–4)	*n* = *215*	*n* = *183*	− 0.01	0.5 (0.1)	1.7 (0.1)	1.1 (0.1)	0.6	< 0.001
Knowledge about condoms (# correct 0–5)	*n* = *243*	*n* = *203*	− 0.02	0.4 (0.1)	4.1 (0.1)	3.5 (0.1)	0.6	< 0.001
Knowledge about consent (# correct 0–5)	*n* = *219*	*n* = *177*	− 0.04	0.3 (0.1)	3.5 (0.2)	2.9 (0.2)	0.5	< 0.001
Awareness of birth control methods (# aware, 0–6)	*n* = *211*	*n* = *190*	− 0.02	0.9 (0.1)	5.0 (0.2)	3.0 (0.1)	2.0	< 0.001
Attitudes								
Attitudes toward birth control (scale, 0–4)	*n* = *202*	*n* = *179*	− 0.06	0.04 (0.1)	2.9 (0.0)	2.9 (0.0)	0.0	0.57
Positive attitudes toward condoms (% positive)	*n* = *217*	*n* = *191*	− 0.06	2.7 (1.8, 4.0)	84.7%	67.1%	17.6%	< 0.001
Self-efficacy								
Confidence stating and asking for consent (% confident)	*n* = *215*	*n* = *185*	0.01	2.1 (1.2, 3.7)	83.3%	70.5%	12.8%	0.02
Confidence discussing sex, contraception (scale 0–4)	*n* = *217*	*n* = *185*	− 0.04	0.3 (0.1)	3.3 (0.1)	3.1 (0.1)	0.2	< 0.001
Confidence to set limits around sexual behavior (scale 0–4)	*n* = *209*	*n* = *176*	− 0.03	0.1 (0.1)	3.4 (0.1)	3.3 (0.1)	0.1	0.12
Definitely know where to get birth control (% confident)	*n* = *225*	*n* = *192*	− 0.03	4.4 (2.8, 7.0)	80.9%	48.9%	32.0%	< 0.001
Confidence going to get contraception (% confident)	*n* = *213*	*n* = *182*	0.01	1.5 (0.9, 2.7)	84.2%	77.6%	6.6%	0.14

There were no significant differences between El Camino and control participants in sexual behavior outcomes, including no penile-vaginal sex ever (OR = 0.7 [0.4, 1.3]), no penile-vaginal sex in the last 3 months (OR = 1.0 [0.5, 1.7]), penile-vaginal sex without any method of contraception in the last 3 months (OR = 1.4 [0.6, 2.9]), or penile-vaginal sex without a condom in the last 3 months (OR = 2.0 [0.7, 5.6]). While not rising to statistical significance, findings for these sexual behavior outcomes appear to favor the control group.

However, findings related to multiple proximal outcomes favor the El Camino group. El Camino participants were almost twice as likely (OR = 1.9 [1.2, 2.9]) as control participants to report that they intended to use condoms if sexually active at post-test (83.1% vs. 72.6%). El Camino participants also had higher summed knowledge scores at post-test compared to control participants across all knowledge domains assessed, including contraception knowledge (*β* = 0.5 [0.1]), method awareness (*β* = 0.9 [0.1]), condom knowledge (*β* = 0.4 [0.1]), and consent knowledge (*β* = 0.3 [0.1]). El Camino participants were almost three times more likely (OR = 2.7 [1.8, 4.0]) to report positive attitudes toward condom use at post-test compared to control participants (84.7% vs. 67.1%). At post-test, El Camino participants were over four times more likely (OR = 4.4 [2.8, 7.0]) than control participants to report that they had the self-efficacy to determine where to go to get contraception for themselves or their partner (80.9% vs. 48.9%). They were more than twice as likely (OR = 2.1 [1.2, 3.7]) to report being confident asking for and stating consent and reported greater confidence about discussing sex and contraception with their partners (*β* = 0.3 [0.1]). No significant impacts were found for intention to use birth control if sexually active, attitudes about birth control, confidence to go to a clinic to get contraception, or confidence around limit setting.

### Moderation

Adjusted estimates (OR [95% CI] or standardized *β* coefficients [SE]) are presented in supplemental Appendix C.

#### Moderation by Gender

Gender moderated the association between the El Camino intervention and confidence in stating and asking for consent (*p* = 0.02) and intention to use contraception at post-test (*p* = 0.001). Among females, El Camino participants were over three times more likely to report that they were confident in stating and asking for consent at post-test compared to control participants (OR = 3.2 [1.3, 7.9]). Among males, no significant association was found (OR = 1.3 [0.6, 2.6]). Among males, El Camino participants were almost three times more likely to report that they intend to use or have their partner use contraception at post-test compared to control participants (OR = 2.7 [1.8, 4.0]). Among females, no significant association was found (OR = 0.8 [0.4, 1.4]).

#### Moderation by Mode of Programming

Mode of programming (in-person vs. virtual) moderated the association between the El Camino intervention and knowledge about birth control (*p* = 0.003), confidence to state and ask for consent (*p* = 0.01), and knowing where to get birth control (*p* = 0.001). While El Camino participants in both virtual and in-person implementation settings had significantly higher birth control knowledge scores at post-test compared to control participants, the intervention effect was stronger for in-person participants. Among those who were enrolled in the program during virtual implementation, El Camino participants had an average of *β* = 0.4 [0.1]) higher knowledge scores at post-test compared to control participants, while El Camino participants who were enrolled in the program during in-person implementation had an average of *β* = 0.6 [0.1]) higher knowledge scores at post-test compared to control participants. El Camino participants enrolled in the program during in-person implementation were almost three times more likely to report that they felt confident stating and asking for consent at post-test compared to control participants (OR = 2.8 [1.5, 5.3]), while there was no significant intervention effect among those enrolled during virtual implementation (OR = 0.7 [0.3, 1.7]). Finally, there was an intervention effect on knowing where to go to get birth control among participants enrolled during in-person implementation, but not for those enrolled during virtual implementation. El Camino participants enrolled in the in-person curriculum were more than six times more likely to report knowing where to go to get birth control at post-test compared to control participants (OR = 6.2 [3.8,10.3]), whereas no significant intervention effect was found among those enrolled in the virtual curriculum (OR = 2.3 [0.9, 6.0]).

#### Moderation by School Type

Type of school (wellness vs. non-wellness center) moderated the association between the El Camino intervention and condom knowledge (*p* = 0.002) and intention to use condoms (*p* = 0.001). While El Camino was associated with higher condom knowledge across school types at post-test, the effect was stronger among El Camino participants attending non-wellness center schools (*β* = 0.5 [0.1]) than wellness center schools (*β* = 0.3 [0.1]). Among wellness center schools, El Camino participants were more likely to report intending to use condoms if sexually active compared to control participants (OR = 2.3 [1.3, 4.2]), whereas no significant intervention effect was found among non-wellness school participants (OR = 1.4 [0.7, 2.7]).

## Discussion

This study presents short-term results from an evaluation of the school-based El Camino program, created for use with Latino youth. It extends previous research on school-based teen pregnancy prevention efforts by assessing the impact of a program for a unique sample of largely recent immigrant and Spanish-speaking youth and examining the role of potential demographic and implementation moderators to better assess implementation context.

Consistent with other adolescent pregnancy prevention evaluations (Chinman et al., 2018; Juras et al., 2019, 2022), this study was underpowered to detect significant differences in sexual behavior outcomes, which may be due to the low prevalence of sexual activity and a short follow-up period. However, the evaluation found substantial intervention impacts on many proximal outcomes cited as key precursors to longer-term behavior change in the program’s theory of change. El Camino participants reported significantly increased knowledge about contraception, condoms, and consent, and increased self-efficacy evidenced by increased confidence in discussing sex with a partner, increased confidence in asking for and giving consent, and knowing where to get birth control. El Camino participants were also significantly more likely than control participants to report they intended to use condoms if sexually active and were more likely to report positive attitudes about condoms. Similar impacts on knowledge, attitudes, intentions, and self-efficacy have been demonstrated in other teen pregnancy prevention evaluations (Chinman et al., 2018; Cole et al., 2024; Manlove et al., 2021), even in the absence of detectable program impacts on sexual behavior outcomes (Chinman et al., 2018; Manlove et al., 2021). These intervention effects are very important in the short-term and may lead to improved contraceptive use and reduced unintended pregnancy in the longer-term. Previous research has demonstrated that greater knowledge (Guzzo & Hayford, 2018) and self-efficacy (Hamidi et al., 2018) each predict greater contraceptive use and a reduced risk of unplanned pregnancy. Specifically, individuals who are aware of contraceptive methods and have knowledge of their benefits and correct use may be more likely to use them and use them correctly, which reduces the risk of unplanned pregnancies and sexually transmitted infections (Guzzo & Hayford, 2018; Hamidi et al., 2018). Additionally, individuals with greater confidence to ask for and state consent may be less likely to have unwanted sex, which is associated with unprotected sex (Edison et al., 2022).

The lack of group differences in self-efficacy to go to a clinic or to set limits around sexual behavior may be due, in part, to ceiling effects. Almost 65% of the sample at baseline reported feeling confident to go to a clinic for contraception, and 71.8% of the sample reported feeling confident to set limits around sexual behavior. Because other reproductive and sexual health interventions have found differences in effects by gender (Juras et al., 2019), we tested whether gender may moderate some of the intervention effects. We found that El Camino had a significant effect on improving female students’ confidence in stating and asking for consent, but this effect was not seen in males. It is possible that confidence in stating and asking for consent is more salient for females than for males. Conversely, male El Camino participants reported a significantly greater intention to use contraception, but this greater intention was not seen in female participants. This finding may be due to there being more room for improvement around contraception intention for males compared to females. Importantly, most of the intervention effects did not vary by gender.

Implementation characteristics have also been found to moderate teen pregnancy program impacts (Juras et al., 2019). The school closures of the COVID- 19 pandemic brought unique challenges and opportunities for the implementation of programming with school-aged youth. As we emerge from the pandemic and into a world where virtual implementation of programming is more common and may offer increased accessibility, it is important to better understand differences between virtual and in-person programming in terms of program impact. In this study, the impacts of the intervention on a range of outcomes were consistently and significantly stronger among participants enrolled in the study during in-person implementation compared to those enrolled during virtual implementation. Because virtual implementation of El Camino was only done during the time of school closures when students were more isolated and generally less engaged, it is impossible to determine the extent to which this finding is more reflective of the time or of the implementation method. However, it is consistent with other recent research finding virtual versions of prevention programming for youth to be less effective (Zervos et al., 2023), and it does offer a caution about implementing sexual and reproductive health programming in a virtual format. Future studies assessing virtual vs. in-person implementation of prevention programming should be conducted to confirm these findings.

This study also found that intervention effects were different depending on whether the intervention was delivered at wellness center schools, where facilitators had more contact with students and ongoing relationships with them, compared to schools without wellness centers, where facilitators fostered new relationships. The intervention effect on higher condom knowledge was stronger for participants in schools without wellness centers; however, impacts on positive attitudes toward birth control and intention to use condoms if sexually active were stronger among wellness center participants. This may be due, in part, to the stronger attendance and youth engagement among intervention participants in wellness center schools given their longer-term relationship with the facilitators in these sites (Faccio et al., 2023). Overall, impacts were seen for students at all schools, and no differences by school type were found for most outcomes.

### Limitations

This study had several limitations to consider in the interpretation of the findings. All data collected were self-report and therefore subject to social desirability and potential underreporting of sexual behavior or overreporting of contraceptive and consent intentions. We attempted to minimize this potential bias by stressing confidentiality at each data collection point and by having students complete and submit questionnaires privately on their own devices. We also had some attrition from our baseline sample as participants were lost to follow-up or uninterested in continuing participation. However, tests of baseline equivalence found no significant differences, other than language, between those who did and did not complete the follow-up.

Our ability to detect intervention impacts on sexual behavior was limited by the short follow-up period. However, research will follow these students for 12 months and test longer-term effects of participation, particularly in sexual behavior. Another important limitation of this study, particularly given a substantial percentage of study participants reporting non-heterosexual attraction, is that sexual behavior outcomes are limited to penile-vaginal sex. While penile-vaginal sexual behavior change is pertinent to teen pregnancy specifically, non-penile-vaginal sexual behavior change may be salient for sexually transmitted infection prevention and other important sexual and reproductive health outcomes among those engaging in other types of sexual behaviors. Future research assessing the program’s impact on other types of sexual behavior is needed. Additionally, intervention participants were more likely to be female and less likely to report only heterosexual attraction compared to control participants, which may have led to an underestimation of program effects given potential lower rates of heterosexual penile-vaginal sex. However, the combination of low and consistent attrition across conditions and a priori randomization reduces the likelihood that baseline inequivalence on these characteristics would bias our findings. Finally, it is possible that participants in the control groups got substantial intervention around relationships and goal setting, known to be associated with sexual health knowledge, attitudes, and behaviors, either from the control curricula itself, or potentially via contamination from intervention participants sharing programming information in the school setting. The intervention benefits received by control group participants, as well as potential intervention contamination, may have limited our ability to detect differences in sexual behaviors between the two conditions, resulting in more conservative estimates of program effects.

While our findings may not be generalizable to other populations and implementation contexts, our study expands the generalizability and external validity of the body of adolescent reproductive and sexual health research to an important population of Latino youth, many of whom are newly arrived in the US. This study demonstrates that newly arrived immigrant youth, who are not yet proficient in English, can benefit from school-based sexual health prevention programming, delivered in person and in students’ preferred language. Other school-based prevention efforts around subjects such as substance use, mental health, and violence may benefit from a similarly culturally and linguistically responsive approach with immigrant youth.

## Conclusions

This study offers important evidence of the short-term impacts of El Camino on key sexual health outcomes for an understudied group of youth. Importantly, these findings not only simply determine intervention effects but also examine key moderators of gender and implementation that offer important information for the replication of this promising prevention program. In sum, El Camino is an innovative school-based sexual health intervention for Latino youth that appears to be effective in improving sexual health knowledge, self-efficacy, and intentions.

## Supplementary Information

Below is the link to the electronic supplementary material.

Supplementary file1 (DOCX 18 KB)

Supplementary file2 (DOCX 31 KB)

Supplementary file3 (DOCX 25 KB)

Supplementary file4 (DOC 217 KB)

## Data Availability

The data presented in this study are not publicly available due to privacy restrictions.

## References

[CR1] Abma, J., & Martinez, G. (2023). Teenagers in the United States: Sexual activity, contraceptive use, and childbearing, 2015–2019. *National Health Statistics Report,* 196. 10.15620/cdc:134957 38170823

[CR2] Akinbami, L. J., Schoendorf, K. C., & Kiely, J. L. (2000). Risk of preterm birth in multiparous teenagers. *Archives of Pediatrics & Adolescent Medicine,**154*(11), 1101–1107. 10.1001/archpedi.154.11.1101 11074850 10.1001/archpedi.154.11.1101

[CR3] Aparicio, E. M., Vanidestine, T., Zhou, K., & Pecukonis, E. V. (2016). Teenage pregnancy in Latino communities: Young adult experiences and perspectives of sociocultural factors. *Families in Society,**97*(1), 50–57. 10.1606/1044-3894.2016.97.3

[CR4] Centers for Disease Control and Prevention. (2023). Youth risk behavior survey. Data summary and trend reports. *Centers for Disease Control and Prevention*. https://yrbs-explorer.services.cdc.gov/. Accessed 8 April 2025.

[CR5] Child Trends. (2021). *El Camino: A goal-setting sexual health promotion program.* https://www.childtrends.org/publications/el-camino-a-goal-setting-sexual-health-promotion-program. Accessed 8 April 2025.

[CR6] Child Trends (2022). *El Camino: Helping teens set life goals and promote sexual health. Fact sheet.* April, 2022. https://cms.childtrends.org/wp-content/uploads/2019/05/ElCaminoFactSheetEnglish_ChildTrends_April2022.pdf. Accessed 8 April 2025.

[CR7] Chinman, M., Acosta, J., Ebener, P., Malone, P. S., & Slaughter, M. E. (2018). A cluster-randomized trial of getting to outcomes’ impact on sexual health outcomes in community-based settings. *Prevention Science: The Official Journal of the Society for Prevention Research,**19*(4), 437–448. 10.1007/s11121-017-0845-6 28971273 10.1007/s11121-017-0845-6 PMC5880746

[CR8] Cole, R., Neelan, T. S., Langan, A., Keating, B., Walzer, J., Asheer, S., & Zief, S. (2024). The impact of making proud choices! on youth’s sexual health attitudes, knowledge, and behaviors. *The Journal of Adolescent Health : Official Publication of the Society for Adolescent Medicine,**74*(4), 787–793. 10.1016/j.jadohealth.2023.10.031 38099897 10.1016/j.jadohealth.2023.10.031

[CR9] Cygan, H. R., McNaughton, D., Reising, V., Fogg, L., Marshall, B., & Simon, J. (2020). Teen pregnancy in Chicago: Who is at risk? *Public Health Nursing,**37*(3), 353–362. 10.1111/phn.12726 32196754 10.1111/phn.12726

[CR10] Edison, B., Coulter, R. W. S., Miller, E., Stokes, L. R., & Hill, A. V. (2022). Sexual communication and sexual consent self-efficacy among college students: Implications for sexually transmitted infection prevention. *The Journal of Adolescent Health,**70*(2), 282–289. 10.1016/j.jadohealth.2021.08.012 34620545 10.1016/j.jadohealth.2021.08.012 PMC9028224

[CR11] Faccio, B., McClay, A., McConnell, K., Gates, C., Finocharo, J., Tallant, J., Martinez, V., & Manlove, J. (2023). Comparing virtual and in-person implementation of a school-based sexual health promotion program in high schools with large Latino populations. *Prevention Science,**24*(Suppl 2), 251–261. 10.1007/s11121-023-01526-0 37351668 10.1007/s11121-023-01526-0 PMC10764389

[CR12] Familias Unidas. (n.d.). Home. Familias Unidas. https://www.familias-unidas.org/. Accessed 8 April 2025.

[CR13] Farb, A., Trivits, L., Oberlander, S., Kelsey, M., Layzer, J., Price, C., & Blocklin, M. (2018). https://aspe.hhs.gov/sites/default/files/migrated_legacy_files//184921/Cuidate_Final_Impact_Report.pdf. Accessed 8 April 2025

[CR14] Fuller, T. R., White, C. P., Chu, J., Dean, D., Clemmons, N., Chaparro, C., Thames, J. L., Henderson, A. B., & King, P. (2018). Social determinants and teen pregnancy prevention: Exploring the role of nontraditional partnerships. *Health Promotion Practice,**19*(1), 23–30. 10.1177/1524839916680797 27913658 10.1177/1524839916680797 PMC5701861

[CR15] Guzzo, K. B., & Hayford, S. R. (2018). Adolescent reproductive and contraceptive knowledge and attitudes and adult contraceptive behavior. *Maternal and Child Health Journal,**22*(1), 32–40. 10.1007/s10995-017-2351-7 28755044 10.1007/s10995-017-2351-7 PMC5764783

[CR16] Hamidi, O. P., Deimling, T., Lehman, E., Weisman, C., & Chuang, C. (2018). High self-efficacy is associated with prescription contraceptive use. *Women’s Health Issues,**28*(6), 509–513. 10.1016/j.whi.2018.04.006 30131220 10.1016/j.whi.2018.04.006 PMC6345511

[CR17] Hans, S. L., & White, B. A. (2019). Teenage childbearing, reproductive justice, and infant mental health. *Infant Mental Health Journal,**40*(5), 690–709. 10.1002/imhj.21803 31318459 10.1002/imhj.21803 PMC6972509

[CR18] Harris, P. A., Taylor, R., Thielke, R., Payne, J., Gonzalez, N., & Conde, J. G. (2009). Research electronic data capture (REDCap)–A metadata-driven methodology and workflow process for providing translational research informatics support. *Journal of Biomedical Informatics,**42*(2), 377–381. 10.1016/j.jbi.2008.08.010 18929686 10.1016/j.jbi.2008.08.010 PMC2700030

[CR19] Harris, P. A., Taylor, R., Minor, B. L., Elliott, V., Fernandez, M., O'Neal, L., McLeod, L., Delacqua, G., Delacqua, F., Kirby, J., Duda, S. N., & REDCap Consortium (2019). The REDCap consortium: Building an international community of software platform partners. *Journal of Biomedical Informatics*, 95, 103208. 10.1016/j.jbi.2019.103208 10.1016/j.jbi.2019.103208 PMC7254481 31078660

[CR20] Juras, R., Tanner-Smith, E., Kelsey, M., Lipsey, M., & Layzer, J. (2019). Adolescent pregnancy prevention: Meta-analysis of federally funded program evaluations. *American Journal of Public Health,**109*(4), e1–e8. 10.2105/AJPH.2018.304925 30789771 10.2105/AJPH.2018.304925 PMC6417595

[CR21] Juras, R., Kelsey, M., Steinka-Fry, K., Lipsey, M., Layzer, J., & Tanner-Smith, E. (2022). Meta-analysis of federally funded adolescent pregnancy prevention program evaluations. *Prevention Science: The Official Journal of the Society for Prevention Research,**23*(7), 1169–1195. 10.1007/s11121-022-01405-0 35841494 10.1007/s11121-022-01405-0

[CR22] Lewin, A., Mitchell, S. J., & Ronzio, C. R. (2013). Developmental differences in parenting behavior: Comparing adolescent, emerging adult, and adult mothers. *Merrill-Palmer Quarterly,**59*(1), 23–49. 10.1353/mpq.2013.0003

[CR23] Lindberg, L. D., Firestein, L., & Beavin, C. (2021). Trends in U.S. adolescent sexual behavior and contraceptive use, 2006–2019. *Contraception: X*, 3, 100064. 10.1016/j.conx.2021.100064 10.1016/j.conx.2021.100064 PMC8102179 33997764

[CR24] Manlove, J., Welti, K., Whitfield, B., Faccio, B., Finocharo, J., & Ciaravino, S. (2021). Impacts of Re: MIX—A school-based teen pregnancy prevention program incorporating young parent coeducators. *Journal of School Health,**91*(11), 915–927. 34553379 10.1111/josh.13078

[CR25] McClay, A., & Moore, K.A. (2022). Preventing births to teens is associated with long-term health and socioeconomic benefits, according to simulation. Child Trends. 10.56417/2270z3088p

[CR26] Morales-Alemán, M. M., & Scarinci, I. C. (2016). Correlates and predictors of sexual health among adolescent Latinas in the United States: A systematic review of the literature, 2004–2015. *Preventive Medicine,**87*, 183–193. 10.1016/j.ypmed.2016.03.005 26972472 10.1016/j.ypmed.2016.03.005 PMC4884463

[CR27] Osterman, M., Hamilton, B., Martin, J. A., Driscoll, A. K., & Valenzuela, C. P. (2021). Births: Final data for 2020. *National Vital Statistics Reports : From the Centers for Disease Control and Prevention, National Center for Health Statistics, National Vital Statistics System,**70*(17), 1–50. 35157571

[CR28] Parekh, J., McClay, A., Faccio, B., Gates, C., Garcia, J., Corryell, A., & Manlove, J. (2021). *Adapting an in-person sexual health program for a virtual setting.* Child Trends. 10.56417/5085e1747r

[CR29] Phipps, M. G., & Nunes, A. P. (2012). Assessing pregnancy intention and associated risks in pregnant adolescents. *Maternal and Child Health Journal,**16*(9), 1820–1827. 10.1007/s10995-011-0928-0 22160612 10.1007/s10995-011-0928-0

[CR30] Rocca, C. H., Doherty, I., Padian, N. S., Hubbard, A. E., & Minnis, A. M. (2010). Pregnancy intentions and teenage pregnancy among Latinas: A mediation analysis. *Perspectives on Sexual and Reproductive Health,**42*(3), 186–196. 10.1363/4218610 20887287 10.1363/4218610 PMC2951312

[CR31] Rosenthal, M. S., Ross, J. S., Bilodeau, R., Richter, R. S., Palley, J. E., & Bradley, E. H. (2009). Economic evaluation of a comprehensive teenage pregnancy prevention program: Pilot program. *American Journal of Preventive Medicine,**37*(6 Suppl 1), S280–S287. 10.1016/j.amepre.2009.08.014 19896030 10.1016/j.amepre.2009.08.014 PMC3020976

[CR32] Rotz D., Dara Lee L., Goesling B., Cook E., Murphy K., & Stevens J. (2016). Final impacts of the teen options to prevent pregnancy program. *Mathematica policy research*. https://www.mathematica.org/publications/final-impacts-of-the-teen-options-to-prevent-pregnancy-program. Accessed 8 April 2025.

[CR33] Vasilenko, S. A., Odejimi, O. A., Glassman, J. R., Potter, S. C., Drake, P. M., Coyle, K. K., Markham, C., Emery, S. T., Peskin, M. F., Shegog, R., Addy, R. C., & Clark, L. F. (2023). Who benefits from school-based teen pregnancy prevention programs? Examining multidimensional moderators of program effectiveness across four studies. *Prevention Science,**24*(8), 1535–1546. 35994193 10.1007/s11121-022-01423-y

[CR34] Welti K. & Faccio B. (2020). Recommendations for developing survey items and outcome measures to evaluate teen pregnancy prevention programs. https://www.childtrends.org/publications/developing-survey-items-and-outcome-measures-to-evaluate-teen-pregnancy-prevention-programs. Accessed 8 April 2025.

[CR35] Xiao, H., Chang, M., Torres, A., Flores, G., & Lau, M. (2023). Preventing teen pregnancy: A qualitative study of the perspectives of parenting and expecting Latino adolescents. *Journal of Pediatric and Adolescent Gynecology,**36*(6), 532–540. 10.1016/j.jpag.2023.07.004 37468034 10.1016/j.jpag.2023.07.004

[CR36] Zervos, A. P., Hensel, D. J., Cope-Barnes, D., James, R., & Ott, M. A. (2023). Effectiveness of youth risk prevention programs when virtually adapted. *The Journal of Adolescent Health,**73*(5), 910–916. 10.1016/j.jadohealth.2023.06.012 37578405 10.1016/j.jadohealth.2023.06.012

