# Supplementary material for: Short-Term Impacts of a School-Based Teen Pregnancy Prevention Program for Latino Youth: a Cluster Randomized Trial
Source: Prev Sci. 2025 Apr 14;26(5):716–26. doi: 10.1007/s11121-025-01805-y (PMC12245966; doi:10.1007/s11121-025-01805-y)
Supplement: Supplementary file 1 — Supplementary file1 (DOCX 18 KB) [file 11121_2025_1805_MOESM1_ESM.docx]

Appendix A.

*Outcome measures, source item(s), and operationalization of outcome measures*

| **Outcome name** | **Source item(s)** | **Constructed measure** |
| --- | --- | --- |
| **Sexual behavior**  Never had penis-in-vagina sex | *Have you ever had penis-in-vagina sex?* | *Dichotomous variable coded 1 if yes, 0 if no and missing otherwise* |
| No penis-in-vagina sex within the last three months | *In the past three months, have you had penis-in-vagina sex, even once?* | *Dichotomous variable coded 1 if yes, 0 if no and missing otherwise* |
| Had penis-in-vagina sex in the last three months without any method of contraception | *In the past 3 months, how often have you or your partner used any method of birth control, including condoms, during penis-in-vagina sex?* | *5 response categories: 4-point scale from “All of the time” (4) to “None of the time” (1) and I don’t know. Coded as 0 if answer is none of the time or I don’t know. Otherwise coded 1* |
| Had penis-in-vagina sex in  the last three months without use of a condom | *In the past 3 months, how often have you or your partner used condoms during penis-in-vagina sex?* | *5 response categories: 4-point scale from “All of the time” (4) to “None of the time” (1) and I don’t know. Coded as 0 if answer is none of the time or I don’t know. Otherwise coded 1* |
| **Intentions** | *Intend to use condoms* | *Intend to use condoms if having sex (dichotomous variable coded as 1 if responds yes definitely vs. 0 if any other response)* |
|  | *Intend to use contraception* | *Intend to use contraception if having sex (dichotomous variable coded as 1 if responds yes definitely vs. 0 if any other response)* |
| **Knowledge** | *Awareness of birth control methods* | *Sum the # of methods responding “yes” to “have you heard of any of these methods of birth control” – response options: 0–6* |
|  | *Knowledge about condoms* | *Sum the # of correct responses to 5 items (sample item: “Condoms have an expiration date”—“don’t know” is considered an incorrect response)* |
|  | *Knowledge about*  *birth control* | *Sum the # of correct responses to 4 items (sample item: “The IUD is more effective at preventing pregnancy than the condom”—“don’t know” is considered an incorrect response)* |
|  | *Knowledge about consent* | *Sum the # of correct responses to 5 items (sample item: “Is someone consenting to have sex with you if they make out with you clothed”—“don’t know” is considered an incorrect response)* |
| **Views & Perceptions** | *Positive attitudes about birth control* | *Average score of 2 items (birth control pills are effective at preventing pregnancy, birth control has too many side effects). Response options on 4-point scale from Strongly disagree to strongly agree)* |
|  | *Positive attitudes about condoms* | *Dichotomous variable measuring 1 if answers “agree or strongly agree” to condoms should always be used, and “disagree or strongly disagree” to it is ok if you forget to*  *use condoms sometimes. Response options on 4-point scale from Strongly disagree to strongly agree* |
| **Self-Efficacy** | *Definitely know where to get birth control* | *Dichotomous variable measured as 1 if respond “definitely” and 0 for all other responses (probably do, probably don’t, or definitely don’t know where to get birth control methods)* |
|  | *Confidence going to a clinic to get contraception* | *Dichotomous variable coded as 1 if “very confident” or “completely confident” on 2 items (vs.*  *all other responses)* |
|  | *Confidence discussing sex, contraception* | *Average score of 2 items (how confident you could talk to your partner about whether or not to have sex, talk to your partner about using condoms. Response options on 4-point scale from “Strongly disagree to strongly agree)* |
|  | *Confidence limit*  *setting* | *Average score of 3 items (sample item: “how confident you could say no to sex if your partner won’t use a condom”) Response options on 4-point scale from “Strongly disagree to strongly agree)* |
|  | *Confidence stating and asking for consent* | *Dichotomous variable coded as 1 if “very confident” or “completely confident” on 2 items (vs.*  *all other responses)* |
