# Supplementary material for: Short-Term Impacts of a School-Based Teen Pregnancy Prevention Program for Latino Youth: a Cluster Randomized Trial
Source: Prev Sci. 2025 Apr 14;26(5):716–26. doi: 10.1007/s11121-025-01805-y (PMC12245966; doi:10.1007/s11121-025-01805-y)
Supplement: Supplementary file 2 — Supplementary file2 (DOCX 31 KB) [file 11121_2025_1805_MOESM2_ESM.docx]

Appendix B.

*Baseline outcome characteristics by treatment condition (n=396-448)*

| Baseline measure | Analytic Sample Size | | | Mean (SD) or % | | | Difference (Mean or %) | Difference (SD units) | P-value |
| --- | --- | --- | --- | --- | --- | --- | --- | --- | --- |
|  | El Camino | Control | Total | El Camino | Control | Total |  |  |  |
| Sexual behavior |  |  |  |  |  |  |  |  |  |
| Never had sex | *n=243* | *n=205* | *N=448* | 71.7% | 72.5% | 72.1% | -0.8% | -0.02 | 0.72 |
| No sexual activity in the last 3 months | *n=240* | *n=204* | *N=444* | 82.0% | 87.2% | 84.4% | -5.2% | 0.09 | 0.50 |
| Sex in the last 3 months without a  method of contraception | *n=230* | *n=196* | *N=426* | 6.0% | 5.2% | 5.6% | 0.8% | -0.03 | 0.76 |
| Sex in last 3 months without a condom | *n=231* | *n=194* | *N=425* | 5.2% | 5.2% | 5.2% | 0.0% | -0.0008 | 1.00 |
| Intentions |  |  |  |  |  |  |  |  |  |
| Intend to use condoms | *n=254* | *n=218* | *N=472* | 64.4% | 63.5% | 63.9% | 0.9% | 0.02 | 0.74 |
| Intend to use contraception | *n=254* | *n=218* | *N=472* | 38.8% | 37.8% | 38.3% | 1.0% | 0.02 | 0.86 |
| Knowledge |  |  |  |  |  |  |  |  |  |
| Knowledge about birth control (#  correct, 0-4) | *n=215* | *n=183* | *N=398* | 0.7 (0.9) | 0.8 (0.9) | 0.7 (0.9) | -0.1 | -0.06 | 0.55 |
| Knowledge about condoms (# correct,  0-5) | *n=243* | *n=203* | *N=446* | 3.0 (1.5) | 3.0 (1.5) | 3.0 (1.5) | 0.0 | -0.03 | 0.69 |
| Knowledge about consent (# correct, 0-  5) | *n=219* | *n=177* | *N=396* | 2.5 (1.8) | 2.6 (1.8) | 2.5 (1.8) | -0.1 | -0.09 | 0.45 |
| Awareness of birth control methods (#  aware, 0-6) | *n=211* | *n=190* | *N=401* | 2.4 (2.1) | 2.3 (2.1) | 2.4 (2.1) | 0.1 | 0.01 | 0.82 |
| Attitudes |  |  |  |  |  |  |  |  |  |
| Attitudes toward birth control (scale 0-  4) | *n=2021* | *n=179* | *N=381* | 2.8 (0.6) | 2.8 (0.6) | 2.8 (0.6) | 0.0 | 0.002 | 0.98 |
| Attitudes toward condoms (% positive) | *n=217* | *n=191* | *N=408* | 75.3% | 76.4% | 75.8 % | -1.1 | -0.02 | 0.80 |
| Self-efficacy |  |  |  |  |  |  |  |  |  |
| Confidence stating and asking for  consent (% confident) | *n=215* | *n=185* | *N=400* | 57.6% | 60.0% | 58.7% | -2.4% | -0.05 | 0.65 |
| Confidence discussing sex,  contraception (scale 0-4) | *n=217* | *n=185* | *N=402* | 2.9 (1.0) | 2.9 (1.0) | 2.9 (1.0) | 0.0 | -0.003 | 0.92 |
| Confidence to set limits around sexual  behavior (scale 0-4) | *n=209* | *n=176* | *N=385* | 3.2 (0.9) | 3.2 (0.9) | 3.2 (0.9) | 0.0 | 0.02 | 0.81 |
| Definitely know where to get birth  control (% confident) | *n=225* | *n=192* | *N=417* | 20.9% | 22.3% | 21.6% | -1.4% | -0.03 | 0.77 |
| Confidence going to get contraception  (% confident) | *n=213* | *n=182* | *N=395* | 66.7% | 62.7% | 64.8% | 4.0% | 0.08 | 0.50 |
