# Supplementary material for: Short-Term Impacts of a School-Based Teen Pregnancy Prevention Program for Latino Youth: a Cluster Randomized Trial
Source: Prev Sci. 2025 Apr 14;26(5):716–26. doi: 10.1007/s11121-025-01805-y (PMC12245966; doi:10.1007/s11121-025-01805-y)
Supplement: Supplementary file 3 — Supplementary file3 (DOCX 25 KB) [file 11121_2025_1805_MOESM3_ESM.docx]

Appendix C.

*Adjusted regression analysis of outcomes by moderator for full outcome measures*

| Outcome | Moderator | Analytic sample size | | Estimate lOR/OR (95% CI) or 𝜷 (SE) | P-Value |
| --- | --- | --- | --- | --- | --- |
|  |  | El Camino | Control |  |  |
| Sexual behavior |  |  |  |  |  |
| *Never had sex* |  | *n=243* | *n=205* |  |  |
|  | Gender |  |  | -0.8 (-1.7, 0.1) | 0.07 |
|  | Mode of programming |  |  | 1.0 (-0.4, 2.4) | 0.15 |
|  | School type |  |  | 0.3 (-1.0, 1.5) | 0.66 |
| *No sexual activity in the last 3 months* |  | *n=240* | *n=204* |  |  |
|  | Gender |  |  | -0.0 (-0.8, 0.8) | 0.95 |
|  | Mode of programming |  |  | 0.7 (-0.5, 2.0) | 0.26 |
|  | School type |  |  | 0.6 (-0.5, 1.8) | 0.29 |
| *Sex in the last 3 months without a method of*  *contraception* |  | *n=230* | *n=196* |  |  |
|  | Gender |  |  | -0.6 (-2.0, 0.7) | 0.38 |
|  | Mode of programming |  |  | -0.6 (-2.3, 1.2) | 0.50 |
|  | School type |  |  | 0.3 (-0.8, 1.5) | 0.54 |
| *Sex in the last 3 months without a condom* |  | *n=231* | *n=194* |  |  |
|  | Gender |  |  | 1.0 (-0.5, 2.4) | 0.19 |
|  | Mode of programming |  |  | 0.7 (-0.6, 2.0) | 0.29 |
|  | School type |  |  | 0.6 (-0.8, 2.0) | 0.39 |
| Intentions |  |  |  |  |  |
| *Intend to use condoms* |  | *n=254* | *n=218* |  |  |
|  | Gender |  |  | -0.7 (-1.5, 0.1) | 0.06 |
|  | Mode of programming |  |  | -0.3 (-1.1, 0.5) | 0.42 |
|  | School type |  |  | 1.8 (0.1, 3.6) | 0.04 |
| *Intend to use contraception* |  | *n=254* | *n=218* |  |  |
|  | Gender |  |  | 1.1 (0.5, 1.8) | 0.001 |
|  | Mode of programming |  |  | -0.1 (-0.6, 0.4) | 0.59 |
|  | School type |  |  | 0.5 (-1.0, 2.0) | 0.51 |
| Knowledge |  |  |  |  |  |
| *Knowledge about birth control (# correct, 0-4)* |  | *n=215* | *n=183* |  |  |
|  | Gender |  |  | -0.1 (-0.3, 0.2) | 0.57 |
|  | Mode of programming |  |  | 0.3 (0.1) | 0.003 |
|  | School type |  |  | 0.1 (-0.4, 0.1) | 0.49 |
| *Knowledge about condoms (# correct ,0-5)* |  | *n=243* | *n=203* |  |  |
|  | Gender |  |  | -0.2 (-0.6, 0.2) | 0.38 |
|  | Mode of programming |  |  | 0.0 (-0.4, 0.5) | 0.85 |
|  | School type |  |  | -0.3 (0.1) | 0.002 |
| *Knowledge about consent (# correct, 0-5)* |  | *n=219* | *n=177* |  |  |
|  | Gender |  |  | -0.2 (-0.7, 0.3) | 0.42 |
|  | Mode of programming |  |  | 0.2 (-0.3, 0.6) | 0.41 |
|  | School type |  |  | -0.5 (-1.1, 0.0) | 0.07 |
| *Awareness of birth control methods (# aware, 0-6)* |  | *n=211* | *n=190* |  |  |
|  | Gender |  |  | -0.1 (-0.6, 0.4) | 0.81 |
|  | Mode of programming |  |  | 0.2 (-0.2, 0.6) | 0.26 |
|  | School type |  |  | 0.5 (-0.1, 1.1) | 0.12 |
| Attitudes |  |  |  |  |  |
| *Attitudes toward birth control (scale, 0-4)* |  | *n=202* | *n=179* |  |  |
|  | Gender |  |  | -0.0 (-0.2, 0.2) | 0.85 |
|  | Mode of programming |  |  | 0.2 (-0.0, 0.5) | 0.06 |
|  | School type |  |  | 0.5 (-0.1, 1.1) | 0.13 |
| *Positive attitudes toward condoms (% positive)* |  | *n=217* | *n=191* |  |  |
|  | Gender |  |  | -0.1 (-1.0, 0.7) | 0.76 |
|  | Mode of programming |  |  | 0.4 (-0.1, 1.0) | 0.13 |
|  | School type |  |  | -0.6 (-1.4, 0.3) | 0.21 |
| Self-efficacy |  |  |  |  |  |
| *Confidence stating and asking for consent (%*  *confident)* |  | *n=215* | *n=185* |  |  |
|  | Gender |  |  | -1.1 (-2.0, -0.2) | 0.02 |
|  | Mode of programming |  |  | -1.0 (-1.9, -0.1) | 0.04 |
|  | School type |  |  | 0.7 (-0.3, 1.7) | 0.17 |
| *Confidence discussing sex, contraception (scale 0-4)* |  | *n=217* | *n=185* |  |  |
|  | Gender |  |  | 0.0 (-0.2, 0.3) | 0.79 |
|  | Mode of programming |  |  | -0.1 (-0.2, 0.1) | 0.50 |
|  | School type |  |  | -0.0 (-0.4, 0.3) | 0.90 |
| *Confidence to set limits around sexual behavior (scale*  *0-4)* |  | *n=209* | *n=176* |  |  |
|  | Gender |  |  | 0.2 (0.0, 0.4) | 0.06 |
|  | Mode of programming |  |  | 0.1 (-0.1, 0.2) | 0.35 |
|  | School type |  |  | 0.0 (-0.3, 0.4) | 0.76 |
| *Know where to get birth control (%*  *confident)* |  | *n=225* | *n=192* |  |  |
|  | Gender |  |  | -0.1 (-0.7, 0.5) | 0.64 |
|  | Mode of programming |  |  | 0.6 (0.2, 1.0) | 0.001 |
|  | School type |  |  | 0.7 (-0.1, 1.4) | 0.09 |
| *Confidence going to get contraception (% confident)* |  | *n=213* | *n=182* |  |  |
|  | Gender |  |  | 0.4 (-0.2, 1.0) | 0.17 |
|  | Mode of programming |  |  | -0.5 (-1.5, 0.4) | 0.29 |
|  | School type |  |  | 1.0 (-0.1, 2.1) | 0.08 |
